# Effects of an Anti-Fertility Product on Reproductive Structures of Common Vole Males and Residues of Compounds

**DOI:** 10.3390/biology13060450

**Published:** 2024-06-19

**Authors:** Kyra Jacoblinnert, Marion Reilly, Raul Da Costa, Detlef Schenke, Jens Jacob

**Affiliations:** 1 Julius Kuehn-Institute, Federal Research Institute for Cultivated Plants, Institute for Epidemiology and Pathogen Diagnostics—Rodent Research, 48161 Muenster, Germanyjens.jacob@julius-kuehn.de (J.J.); 2 Department of Behavioral Biology, University of Osnabrueck, 49076 Osnabrueck, Germany; 3 Centre for Reproductive Medicine and Andrology, University of Muenster, 48149 Muenster, Germany; rauldacosta@outlook.de; 4 Julius Kuehn-Institute, Federal Research Centre for Cultivated Plants, Institute for Ecological Chemistry, Plant Analysis and Stored Product Protection, 14195 Berlin, Germany; detlef.schenke@julius-kuehn.de

**Keywords:** fertility control, sperm analysis, common voles, 4-vinylcyclohexene diepoxide, triptolide

## Abstract

**Simple Summary:**

Bait-based fertility control could contribute to rodent pest management. Bait containing 4-vinylcyclohexene diepoxide (VCD) and triptolide (TP), registered as ContraPest^®^, was delivered to male common voles to assess the effects on reproductive structures and residues in the liver and testes. There were no effects or inconclusive effects for most reproductive parameters considered. However, there was an increase in sperm defects in voles treated for 14/28 days and fewer normal sperm cells in voles treated for 28 days. There were no TP residues in the testes, few and low TP residues and no VCD residues in the liver tissue. Treatments with VCD + TP seemed to have minor effects on male reproductive organs.

**Abstract:**

Some rodent species cause significant damage to agriculture and forestry, and some can transmit pathogens to humans and livestock. The common vole (*Microtus arvalis*) is widespread in Europe, and its population outbreaks have resulted in massive crop loss. Bait-based fertility control could contribute to rodent pest management. Bait containing 4-vinylcyclohexene diepoxide (VCD) and triptolide (TP), registered as ContraPest^®^, was delivered to male common voles for 14 or 28 consecutive days. The effects on reproductive structures and residues in the liver and testes were assessed. There was no effect on testis weight, sperm viability, sperm motility and oxidative stress in sperm cells. Results regarding the mitochondrial membrane potential of sperm, DNA fragmentation and progressively motile sperm cells were inconclusive. However, there was an increase in morphological sperm defects in voles treated for 14/28 days and fewer normal sperm cells in voles treated for 28 days. There were no TP residues in the testes, few and low TP residues and no VCD residues in liver tissues, making considerable secondary exposure to non-target species unlikely. Treatments with VCD + TP seemed to have minor effects on the reproductive organs of males. Further studies should evaluate the effect of VCD + TP on females and on the reproductive success of common voles and other pest rodent species.

## 1. Introduction

Fertility control is potentially a more sustainable and humane approach to managing overabundant vertebrate pest species than using lethal methods, and it was first proposed 5 decades ago [1,2]. The ethical judgment of the public is usually not in favor of lethal management methods [3,4], resulting in increasing interest in fertility control as an alternative. Ideally, an anti-fertility agent is easily delivered to an appropriate proportion of the population that is also species-specific, renders the target species infertile for the duration required, has no or minimal side effects and is environmentally safe and economically viable [5]. However, only a small number of anti-fertility compounds meet these criteria, and only a few products are registered [6].

The liquid anti-fertility bait ContraPest^®^ is registered in the USA to manage Norway rat (*Rattus norvegicus,* Berkenhout, 1769) and house mouse (*Mus musculus*, Linnaeus, 1758) populations. It contains two complementary ingredients, VCD and TP, that target the male as well as the female reproductive system [7]. VCD induces early menopause and is, therefore, often used as a model compound for ovotoxicity. It depletes primordial and primary follicles [8,9] and increases oxidative stress (ROS), which results in a reduction of epididymal function and spermatogenesis [10]. TP is a plant extract from the roots of the thunder god vine (*Tripterygium wilfordii* Hook. f.) and is used in traditional Chinese medicine [11]. TP is registered in China for rodent management [6]. It reduces sperm motility, sperm number and sperm viability, as well as testis weight and spermatogenesis, and increases structural and morphological changes, such as head–tail separation and degenerated plasma membrane or head deformations in male Norway rats and house mice [12,13,14]. In females, it targets developing follicles and increases the apoptosis of secondary follicles [15]. A combination of VCD and TP is assumed to have additive and more rapid effects in the target species [16]. Nevertheless, some studies indicate a risk of toxic side effects on organs such as the liver, testes and ovaries [17].

At present, there are only two other fertility control compounds besides Contrapest available for use in registered products. A combination of the two synthetic hormones, levonorgestrel (E) and quintestrol (P), named EP-1, can be delivered orally and is registered in Tanzania to control multimammate mouse (*Mastomys natalensis* Smith, 1834) populations. Moreover, for rodents, Gonacon was registered for black-tailed prairie dogs (*Cynomys ludovicianus* Ord, 1815) in the USA, but it needs to be injected [5], which makes it unsuitable for the management of rodent species where individuals cannot be targeted cost-effectively.

The common vole (*Microtus arvalis* Pallas 1778) has, like most small rodent species, a very high reproductive rate and is one of the major vertebrate pest species in Europe [18]. During outbreaks, which occur every 3–5 years, they cause severe agricultural and financial damage. In 2007, a loss of EUR 130 million was related to 11% pre-harvest crop losses [19]. By including grassland and other crops, the financial damage was estimated to reach up to EUR 700 million [19].

Rodents host viruses and other known and unknown pathogens and parasites, as well as several vectors that carry human pathogenic parasites [20]. Therefore, they have the potential to be a source of emerging diseases both in the present and in the future. According to the World Health Organization [21] and Colombe et al. (2019), the number of human zoonotic disease cases related to rodents approaches 400 million a year, causing billions of USD in health costs [22]. Invasive rodent species can also be problematic if they threaten native flora or fauna. However, rodents are important for the ecosystem, so every management needs to consider both the positive and negative effects of the target rodent species [18].

Rodenticides are presently the primary control option to reduce rodent populations, but, like all approaches, they have advantages (easy to use, cheap) and disadvantages (non-target exposure) [23,24]. Accumulation of compounds in target and non-target species, as well as persistence in the environment, are of concern regarding anticoagulant compounds because the resulting exposure of non-target species and other compartments of the environment is common [25,26,27]. Burrow baiting, or the use of bait boxes, indoor applications and the removal of carcasses, might help to mitigate the risks [24].

Due to the ecotoxicological risks of the large-scale application of rodenticides in agriculture, restrictions by registration authorities are increasingly imposed, and the number of products for agricultural use is decreasing. In the European Union (EU), zinc phosphide is the only rodenticidal bait compound registered [28]. National product registrations in EU member states are restricted to this compound to manage common voles in agriculture [26]. Therefore, suitable alternatives are urgently needed. Fertility control, as part of an ecologically based rodent management approach, might be a sustainable tool to control rodent pest species such as voles [6,23,29]).

Fertility control has been suggested as a suitable means to manage outbreaks of small pest rodents [6]. In contrast to commensal situations where most people have zero tolerance for pest rodents, low densities of common voles are irrelevant to farmers. To prevent outbreak peaks of pest rodents, fertility controls might be sufficient to prevent 1–2 L early in the breeding season [30]. In this study, we assessed the effects of VCD + TP (ContraPest^®^) on the male reproductive system after 14 and 28 d of continuous delivery and on the persistence of the compounds in liver and testis tissue. The results can help to assess the suitability of VCD + TP for common vole management and potential issues related to the residues of these compounds in the target species.

## 2. Materials and Methods

All trials were conducted in 2019–2020 in the animal holding facilities of Julius Kuehn Institute in Münster (51°5829.6 N; 7°3402.2 E), Germany, with common voles captured in the surroundings of Münster and their F_1/2_ offspring. Common voles were housed individually in standard rodent cages (410 × 250 × 150 mm, Dieter Wenzel, Detmold, Germany), with softwood chips and hay as bedding and a polycarbonate shelter. Rodent pellets (Altromin 1324; Altromin Spezialfutter GmbH & Co. KG, Lage, Germany) and tap water were provided ad libitum at all times with a natural light–dark cycle.

### 2.1. VCD + TP Baiting and Sample Preparation

Male common voles were chosen randomly, weighted and grouped into two experimental and two control groups with 10 animals each. All voles in both groups were offered 2.5 mL of a liquid control bait without active ingredients in 15 mL centrifuge tubes with a rubber plug and a sipper for two days. On day 3, the tubes were filled every day with 2.5 mL of either the liquid bait containing 0.09604% VCD and 0.00118% TP (=ContraPest^®^) or with the identical formulation but without the two active ingredients. Trials were conducted for 14 or 28 consecutive days (experimental treatment groups 14/28 days with 10 individuals each and control groups 14/28 days with 10 individuals each). Bait consumption was measured daily. After the baiting period, the voles were euthanized with CO_2_, and testes were removed and stored in phosphate-buffered saline (PBS) to prevent dehydration until all samples were collected. Afterward, fat was removed, and epididymis and testes were weighted with a laboratory scale to the nearest mg (Kern, PCB 2500-2, Kern & Sohn GmbH, Ziegelei 1, Balingen, Germany). Testes and epididymis were separated and stored at −20 °C until further analysis. The epididymis samples were transferred into Eppendorf tubes with 2 mL PBS, and the caput epididymis was minced with scissors three to five times and incubated for 3 h for sperm swim out. Afterward, the epididymal tissue was removed. A sample of liver tissue was collected from all animals that were baited for 28 consecutive days and stored at −80 °C until further analysis.

### 2.2. Evaluation of Sperm DNA Fragmentation

The sperm chromatin structure assay (SCSA) protocol has been described elsewhere by Evenson et al. [31]. Briefly, a total of 1 × 10^6^ cells were treated with a low-pH (1.2) detergent solution for 30 s and then stained with 6 mg/L purified Acridine Orange (AO) in phosphate–citrate buffer, pH 6.0. When excited at 488 nm, AO intercalated with double-stranded DNA emits green fluorescence, and AO associated with single-stranded DNA emits red fluorescence. Non-fragmented (green fluorescence) versus fragmented DNA (red fluorescence) cytograms were used to determine the percentage of DNA fragmentation (DNA fragmentation index (DFI)) of the entire sperm population (%DNA fragmentation index or %DFI), which was quantified based on the increased ratio of red/[red + green] fluorescence. A standard sample (≤5 %DFI) was used as control at the beginning of each round of experiments to verify threshold differentiating non-fragmented DNA spermatozoa (main population of cells) and fragmented spermatozoa (cells outside of the main population).

### 2.3. Simultaneous Evaluation of Sperm Membrane Integrity, Mitochondrial Membrane Potential and ROS Production

A staining consisting of LIVE/DEAD™ Fixable Blue Dead Cell Stain kit (LD) (Invitrogen™ Life Technologies GmbH, Darmstadt, Germany), MitoTracker™ Deep Red FM (MT)) (Invitrogen™ Life Technologies GmbH, Darmstadt, Germany) and CellROX™ Green Reagent (CR) (Invitrogen™ Life Technologies GmbH, Darmstadt, Germany) was performed for the simultaneous evaluation of sperm membrane integrity, mitochondrial membrane potential and reactive oxygen species (ROS) production, respectively. For this purpose, sperm suspensions were incubated in the dark for 30 min at 37 °C with the LD 0.1% + MT 0.02 μM + CR 5 μM. Then, sperm were washed once (300 g, 5 min, 37 °C) to remove the excess dye before being re-suspended in 500 µL of human tubal fluid before measurement by flow cytometry.

### 2.4. Flow Cytometry

Flow cytometry was conducted using a CytoFLEX S (Beckman Coulter, Krefeld, Germany) flow cytometer equipped with four diode lasers emitting at 375, 405, 488 and 638 nm, along with 15 optical filters. For SCSA, the dye was excited with a 488 nm laser and two different filters, 525/40 BP and 690/50 BP, were used to detect green and red fluorescence, respectively. In the case of the simultaneous evaluation of sperm membrane integrity, mitochondrial membrane potential and ROS production, LD dye was exited using a near UV laser 375 nm and its corresponding emitted fluorescence was detected with a 450/45 BP filter. For the MT, the dye was excited with a red 638 nm laser and the emitted fluorescence was collected with a 660/20 BP filter. The CR dye was excited with a blue 488 nm laser, and the emitted fluorescence was collected using a 585/42 BP filter.

Fluorescence information regarding 10,000 spermatozoa was collected for each sample/condition. Unstained and single-stained control samples were used to set quadrants and determine background fluorescence. Additional positive controls such as Triton X-100 0.1% permeabilized spermatozoa and UVB 312 nm light exposed spermatozoa 30 min were also considered for the setting of the quadrants. For the triple staining, positive controls such as Triton X-100 0.1% permeabilized spermatozoa (for LD), a mitochondrial membrane potential (MMP) disruptor, carbonyl cyanide 3-chlorophenylhydrazone (CCCP), 50 µM (for MT) and a pro-radical, H_2_O_2_ 200 µM (for CR) were also used for setting the quadrants. Files were exported and analyzed using FCS Express 6 Plus Research Edition (FCS Express V 6; De Novo Software, Pasadena, CA, USA).

### 2.5. Sperm Morphology and Motility

Sperm morphology was assessed by fixing and staining air-dried smears of the samples with Diff Quick solution (Diagonal, No.10736131). For each sample, 2 × 200 sperm were evaluated under a light microscope (Leitz Laborlux S, Leica Microsystems GmbH, Wetzlar, Germany) using 40 × 10 magnification. Spermatozoa were classified as having normal shape or defects (head, mid-piece or tail defects) and categorized into one, two or more than three defects for each sperm cell. Results stated are percentages for each classification.

For quantitative sperm motility measurement, 20 µL of the sample was pipetted on a microscope slide and, if necessary, diluted with PBS. Two hundred sperms were immediately subjectively counted with a digital blood cell counter (Counter AC-12, Hecht Glaswarenfabrik GmbH & Co KG, Sondheim, Germany) under a light microscope and grouped into non-motile (NM), motile (M) and progressively motile (PM) sperm. Results stated are percentages.

### 2.6. Triptolide Residues in Liver and Testicle Samples

The method for the analysis of triptolide in liver and testicle samples was inspired by Xue et al. and Wei et al. and adapted to the current situation [32,33]. Triptolide (CAS-No: 38748-32-2), triptolide-d3 (surrogate, Biozol, Eching, Germany) and atrazine-d5 (internal standard) were delivered from LGC Standards (www.lgcstandards.com). The solvents ethyl acetate (for residue analysis), methanol (for LCMS) and acetonitrile (for LCMS) were all Chemsolute, a brand of Th. Geyer (www.thgeyer.com). The quechers extract pouch salt mixture of sodium chloride and magnesium sulfate (1:4) was purchased from Agilent Technologies (Waldbronn, Germany), and ammonium fluoride p.a. was purchased from Carl Roth (Karlsruhe, Germany). Deionized water was produced in-house with Arium 611UV (Sartorius, Goettingen, Germany). The stock solutions (1 mg/mL) and working and calibration standards were in acetonitrile and stored at −20 °C. Samples were stored at −80 °C until analysis.

Liver (mean value 0.46 ± 0.29 g wet weight (ww)) and testis (mean value 0.13 ± 0.05 g ww) samples were weighed in a falcon tube and spiked with the surrogate triptolide-d3. After 5 min, the sample was homogenized in ethyl acetate/water (2:1; *v*/*v*) using an Ultra Turrax T25 (IKA, Staufen, Germany). With the addition of 5 g of Quechers salt mixture and the subsequent ultrasonic treatment (Sonorex Super 10P, Bandelin, Berlin, Germany), triptolide was converted in the organic phase. After centrifugation (5000 rpm for 10 min at room temperature, Heraeus Megafuge 16 R, Thermo Fisher Scientific, Waltham, MA, USA), an aliquot of the supernatant was evaporated to dryness under a nitrogen stream. The residue was redissolved in acetonitrile/water (1:1; *v*/*v*), including the internal standard atrazine-d5, and briefly vortexed. The solution was filtrated through a ROTILABO syringe filter (PTFE, 0.2 µm, Karlsruhe, Germany) in an autosampler vial (Table S1). Liquid chromatography–electrospray tandem mass spectrometry (LC-ESI-MS/MS; 1290 Infinity II, Agilent, Technologies, Santa Clara, CA, USA and QTRAP 6500+, SCIEX, Framingham, MA, USA) was used to measure the TP content in the samples. The instrument settings for the chromatographic separation and the operating parameters of the positive electrospray ionization mode of the mass spectrometer are stated in Tables S2 and S3. The identification and quantification were conducted with a characteristic precursor (Q1)—product ion (Q3)—transition Triptolide 1. The conformation of the identity of triptolide was obtained with the transition Triptolide 2 (Table S3). Results were additionally verified by recording an enhanced product ion spectrum in the ion trap mode of the mass spectrometer with dynamic filling time (Table S4). The threshold for acceptance of triptolide was a match of more than 80% between the enhanced product ion spectra of the sample and corresponding standards. The concentrations of triptolide were calculated with peak areas with Analyst 1.7.1.

The calibration curves for triptolide and triptolide-d3 were linear with r^2^ > 0.99 over the whole range (0.2–20 pg/µL). The analytical procedure was confirmed by recovery tests with blank pig liver (Table S5). All trail testis and liver samples were spiked with the surrogate triptolide-d3 for ongoing validation of the analytical performance (Table S6). All samples were measured twice. The reporting limit refers to the lowest calibration level with a signal-to-noise ratio > 6:1 and a relative standard deviation < 20% in the sequence. The reporting limit for trial liver samples was 2 ng/g ww, and for testis samples, it was 1 ng/g ww. The measured concentrations of triptolide were neither surrogate nor recovery corrected.

### 2.7. 4-Vinylcyclohexene Dioxide Residues in Liver Samples

The analysis of 4-vinylcyclohexene dioxide in liver samples was based on ideas by Keller et al., Fontaine et al. and Chiappe et al. and adapted to the current sample situation [34,35,36]. 4-vinylcyclohexene dioxide (CAS-No: 106-87-6), cis-cyclodecene, piperitone (surrogate) and 2-cyclohexen-1-one (internal standard) (Sigma-Aldrich, Taufkirchen, Germany) were used. The solvent acetonitrile (for LCMS) was all Chemsolute, a brand of Th. Geyer. Sodium chloride was purchased from Carl Roth (Karlsruhe, Germany). Deionized water was produced in-house with Arium 611UV (Sartorius). The stock solutions (1 mg/mL) and working and calibration standards were in acetonitrile and stored at −20 °C. Samples were stored at −80 °C until analysis.

Liver (mean value 0.43 ± 0.14 g ww) samples were weighed into a falcon tube and spiked with the surrogate cis-cyclodecene and piperitone. After 5 min, the sample was homogenized for 1 min in acetonitrile using an Ultra Turrax, followed by an ultrasonic treatment for 10 min, both with ice cooling. After centrifugation (5000 rpm for 10 min, Heraeus Megafuge 16 R) at room temperature, an IS solution was added to an aliquot of the supernatant and briefly vortexed. The solution was filtrated through a ROTILABO syringe filter (PTFE, 0.2 µm) in an autosampler vial (Table S7).

Analyses were performed using gas chromatograph and a connected mass spectrometer (Trace GC Ultra + TSQ Quantum GC XLS (Thermo Scientific, Dreieich, Germany)). The instrument settings for the chromatographic separation and the operating parameters of the negative ion chemical ionization mode of the mass spectrometer are stated in Table S8. The identification and quantification took place with the sum of characteristic masses from 4-vinylcyclohexene dioxide detected on quadrupole 1 (Q1) (Table S9). The calibration curves for 4-vinylcyclohexene dioxide and the surrogates were linear with r^2^ > 0.99 over the whole range (50–3000 pg/µL). The analytical procedure was validated by recovery tests with blank calf liver. All trail liver samples were spiked with the surrogate cis-cyclodecene and piperitone for ongoing validation of the analytical performance (Table S10). All samples were measured twice. The reporting limit refers to the lowest calibration level with a signal-to-noise ratio > 6:1 and relative standard deviation <20% in the sequence. The reporting limit for trial liver samples was 200 ng/g ww. The measured concentrations were neither surrogate nor recovery corrected.

### 2.8. Statistical Analysis

A Welch test was conducted in SPSS version 28 (IBM Corp.; released 2021; IBM SPSS Statistics for Windows, Version 28.0; Armonk, NY, USA: IBM Corp.) to test for effects of treatments on the parameters high DFI, + ROS, −MMP, sperm viability, sperm motility and sperm morphology). If the Welch test indicated a statistically significant difference, a one-way analysis of variance (ANOVA) with post hoc test (Tukey test) was conducted.

## 3. Results

### 3.1. Testis Weight and Bait Consumption

Testis weight per body weight (C 14 = 0.7, T 14 = 0.7, C 28 = 0.66, T 28 = 0.54 weight/bw in %) did not differ significantly among control and treatment groups (*p* = 0.564). Furthermore, no difference in bait consumption between the 14- and 28-day control- and treatment groups (14-day: *p* = 0.911, 28-day *p* = 0.076) was observed.

### 3.2. Flow Cytometry

**Figure 1:**
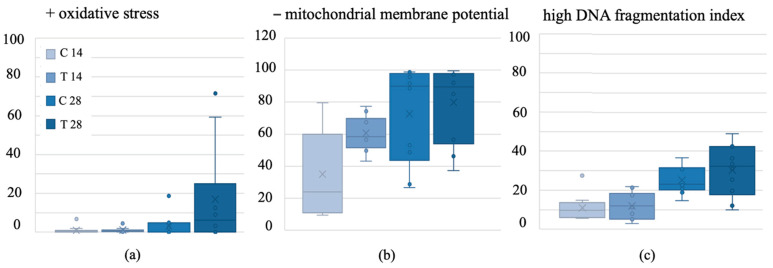
(**a**) Oxidative stress in common vole sperm after a 14- or 28-day baiting period with either a control bait or an anti-fertility bait containing VCD + TP. (**b**) Negative mitochondrial membrane potential of common vole sperm after a 14- or 28-day baiting period with either a control bait or an anti-fertility bait containing VCD + TP. (**c**) High DNA fragmentation index of common vole sperm after a 14- or 28-day baiting period with either a control bait or an anti-fertility bait containing VCD + TP. VCD = 4-vinylcyclohexene diepoxide; TP = triptolide; C = control group; T = treatment group; box = 25/75% quartile; X = mean; horizontal line = median; whiskers = minimum/maximum; dots = outliers exceeding 1.5-fold interquartile distance).

There were no statistically significant treatment effects regarding the viability of sperm (*p* = 0.519). ROS was generally low, and there was no treatment effect on ROS (Figure 1a) (*p* = 0.249). −MMP was 69% higher in the 28-day treatment than in the 14-day treatment (*p* = 0.000) (Figure 1b) but similar to all other groups. Similar results were found for high DFI, which was also higher in the 28-day treated group than in all other groups, especially in the 14-day treatment group (*p* = 0.008) (Figure 1c).

### 3.3. Morphology

**Figure 2:**
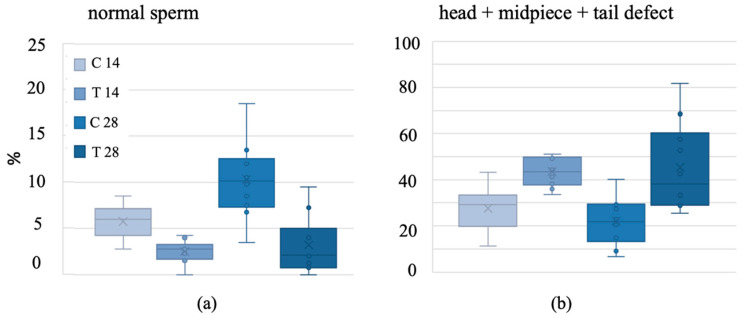
(**a**) Normal sperm morphology [%] of common vole sperm cells and (**b**) head, midpiece and tail defects in common vole sperm cells after a 14- or 28-day baiting period with either a control bait or an anti-fertility bait containing VCD + TP. VCD = 4-vinylcyclohexene diepoxide; TP = triptolide; C = control group; T = treatment group; box = 25/75% quartile; X = mean; horizontal line = median; whiskers = minimum/maximum; dots = outliers exceeding 1.5-fold interquartile distance).

The mean percentage of sperm with a normal morphology was <10%. There were <5% normal sperm cells in the 14-day treatment group, which tended to be lower than in the 14-day control group (*p* = 0.063). The percentage of normally shaped sperm cells was 69% lower in the 28-day treatment group than in the 28-day control group (*p* < 0.001), but there was no difference between both treatment groups (*p* = 0.944) (Figure 2a). There were 57% (14-day treatment) and 105% (28-day treatment) more sperm cells with three or more morphological defects in both treatment groups than in their control groups (14-day treatment: *p* = 0.03; 28-day treatment: *p* = 0.01) (Figure 2b), but there was no difference between treatment groups (*p* = 0.989).

### 3.4. Motility

**Figure 3:**
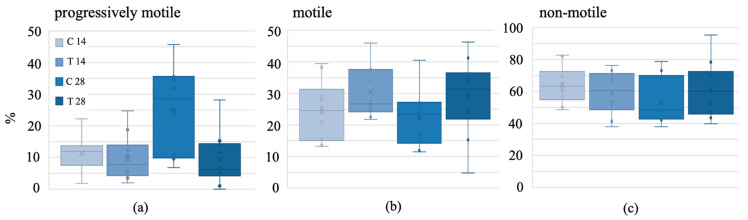
(**a**) Progressively motile, (**b**) motile and (**c**) non-motile sperm cells [%] of common voles after a 14- or 28-day baiting period with either a control bait or an anti-fertility bait containing VCD + TP. VCD = 4-vinylcyclohexene diepoxide; TP = triptolide; C = control group; T = treatment group; box = 25/75% quartile; X = mean; horizontal line = median; whiskers = minimum/maximum; dots = outliers exceeding 1.5-fold interquartile distance.

There was a statistically significantly lower percentage of progressively motile sperm in the 28-day treatment group (9%) than in the 28-day control group 25% (*p* = 0.005) (Figure 3a). The percentage of progressively motile sperm was similar between the 14-day and 28-day treatment group (*p* = 1.0) and the 14-day treatment and control groups (*p* = 0.984) (Figure 3). There was no difference between groups regarding the percentage of motile and non-motile sperm (*p* > 0.05) (Figure 3b,c).

### 3.5. VCD + TP Residues in Liver Tissue, TP Residues in Testis Tissue

There were no VCD residues detected in the liver tissues of common voles treated for 14/28 days, and there were no residues in control animals that consumed bait without the compounds for 28 days. TP residues in liver tissue were present in 2 of 10 common voles treated for 28 days (2 ng/g and 22 ng/g). Triptolide was absent from testis tissues of 14-day and 28-day treatment voles and control voles.

## 4. Discussion

This is the first study of the reproductive effects of VCD + TP in a species of the family Cricetidae, which comprises about 680 species [37]. The main results indicate that a VCD + TP treatment for 14 and 28 days does not affect testis weight, percentage of live sperm cells, ROS and general motility of sperm cells. The 28-day treatment slightly decreased the percentages of normal sperm cells as well as of progressively motile sperm cells, and it clearly increased the number of sperm defects.

Prior work has documented the effectiveness of VCD and TP on the reproductive system in two species of the family Muridae: male Norway rats and house mice. Both compounds seem to have additive effects, which lead to follicle depletion and reduction in sperm quality [7,8,9,11,15,16]. However, mechanisms and effects can differ among taxa. It is possible that there are fundamental differences between species of the family Muridae, such as Norway rats and house mice, and the family Cricetidae, such as common voles.

A comparison of the results of this study with published work is difficult because of variations in doses administered and treatment duration. The findings of Ni et al. that reported that the epididymis and testis weight of Sprague Dawley rats decreased significantly after a daily gastric infusion of 100, 200 and 400 µg/kg TP for 8 weeks [38] was not confirmed. A longer treatment period might decrease testis weight in common voles. In common voles, there was no indication of treatments affecting common vole sperm mobility (total mobility, progressive mobility, immobility) despite reports that the motility of rat sperm cells decreased to zero after TP treatment [38]. Measures of progressively motile sperm cells in our study were of considerable variation for unknown reasons that may be balanced with a higher level of replication.

Triptolide affects the viability of sperm cells [39], reduces mitochondrial membrane potential and increases oxidative stress (ROS) in laboratory rats and mice [40]. In common voles, there was no difference in sperm cell viability and +ROS among treatments and controls. However, there was higher variation in the percentage of living sperm cells and in ROS in animals that were treated with VCD and TP for 28 consecutive days, indicating treatment effects in some animals. One possible explanation for such high variation might be that we conducted our experiments with wild voles and their offspring. Individuals of the same species from the natural environment can differ in their responses in contrast to well-standardized laboratory strains [41]. However, for the future application of an anti-fertility bait, wild animals are the target, and therefore, variations in individual parameters are likely to matter. In this first study with common voles, a reasonable sample size was used that reflects the level of replication (*n* = 6–13 per group) in previous studies with wild rodents [7,42,43]. Future trials can benefit from our results as they allow us to adjust sample size according to now-known effect sizes.

We did find group differences in −MMP and DFI but not in a consistent pattern that indicates clear treatment effects. The bait contains a high concentration of sugar [44], which is often used to increase the palatability of baits but is also known to negatively affect sperm quality in Wistar rats [45,46,47].

Our results provide compelling evidence for an increase in morphological defects like head–tail separations, head, midpiece or tail deformations after 14 as well as after a 28-day treatment with VCD + TP. This is supported by the findings of Huynh et al. [14] and indicates that sample size was sufficient for this parameter despite variability. Nonetheless, the proportion of deformed sperm did not increase with the duration of the treatment, and hence, there was an increase in the total dose delivered to common voles. From this point of view, on the one hand, it might not be necessary to offer bait for more than 14 days to achieve the effect observed. On the other hand, it may be necessary to deliver bait for much longer to achieve higher sperm disruption. Witmer et al. observed a 4-month infertility in rats after 50 days of VCD and TP treatment [42]. In addition to the duration of treatment, the daily dose of TP + VCD delivered to the target animals should matter for treatment effects. However, it is not known what effect size in males is required to inhibit or decrease the reproductive success of common voles using an anti-fertility agent. It is assumed that effects on sperm cells need to be drastic because only a limited number of functioning sperm cells are necessary for fertilization.

Ni et al. reported that TP accumulates in testis tissue but stated that further studies are needed to elucidate if the residues are active metabolites [38]. After 28 days of treatment, no accumulation of TP in testis tissue has been observed. Merely two animals had very minor TP residues in liver tissue. VCD residues were not present in liver tissue. Given that samples were collected on the last day of treatment, there should be even fewer residues present later because TP and VCD are metabolized quickly—at least in SD and Wistar rats (for TP) and Fisher rats (VCD) [48,49,50,51]. Based on this and our results for VCD and TP residues, we conclude that VCD and TP residues are unlikely to be transferred to non-target species via secondary exposure. Further studies with a larger sample size might be beneficial in investigating the toxicity of VCD + TP in common voles.

## 5. Conclusions

Generally, the impacts of VCD + TP at the concentrations used in the registered product ContraPest^®^ on male reproductive organs, as well as sperm quantity and quality, seem minor. This may raise doubt that these effects alone can substantially reduce reproductive output to such a degree that outbreak amplitudes of common voles are dampened. Higher concentrations of the two compounds than present in ContraPest^®^ should be trialed with male/female common voles. Mating experiments are required to assess the effect of VCD + TP on litter size as an ultimate test of the potential of VCD + TP for the management of outbreaks of common voles and possibly other cricetide rodent species. Given the high variation among wild-derived common voles, the sample size used here and in other previous studies (8–10 animals/group) should be increased in future studies.

## Data Availability

Key data are reported in the article and in the Supplementary Materials. Further data presented in this study are available on request from the corresponding author.

## Supplementary Materials

The following supporting information can be downloaded at: https://www.mdpi.com/article/10.3390/biology13060450/s1, Table S1: TP—analysis: Workflow of sample preparation; Table S2: TP—analysis: Configuration of LC-MS/MS; Table S3: TP analysis—LC-MS/MS—MRM-conditions of the analyte, surrogate and internal standard (precursor (Q1) and product ions (Q3) in m/z and declustering potential (DP), entrance potential (EP), collision energy (CE) and cell exit potential (CXP) in V); Table S4: TP—analysis: Confirmation by Enhanced Product Ion spectra; Table S5: TP—analysis: Validation—Recovery (REC) and relative standard deviation (RSD) with pig liver (*n* = 5); in control samples (*n* = 2) all not detected; n. d. (not detected) = < Reporting Limit (RL); Table S6: TP—analysis: Validation—Recovery (REC) of the surrogate triptolide-d3, over all trial liver and testicle samples + relative standard deviation (RSD) with 5 ng/sample wet weight; Table S7: VCD—analysis: Workflow of sample preparation; Table S8: VCD—analysis: Configuration of GC-MS; Table S9: VCD—analysis: GC-MS—retention time and detected masses of analytes used for identification and quantification of the analyte, surrogate and internal standard on quadrupole 1; Table S10: VCD—analysis: Validation—Recovery (REC) and relative standard deviation (RSD) of 4-vinylcyclohexene dioxide and the surrogates cis-cyclodecene and piperitone in calf liver (control samples (*n* = 2) all not detected) for method development. The recovery of the surrogates, over all trial liver samples + relative standard deviation (RSD) were done with 1000 ng/g wet weight.
