# Supplementary material for: Effects of an Anti-Fertility Product on Reproductive Structures of Common Vole Males and Residues of Compounds
Source: Biology (Basel). 2024 Jun 19;13(6):450. doi: 10.3390/biology13060450 (PMC11200569; doi:10.3390/biology13060450)
Supplement: Supplementary file 1 [file biology-13-00450-s001.zip › biology-2962613-supplementary.pdf]

biology

MDPI

Table S1. TP – analysis: Workflow of sample preparation.

Defrosting of liver samples, and weigh in pp - tubes
⇩
Spike with surrogates
⇩
Addition of 10 mL ethyl acetate
5 mL water
⇩
Homogenisation (IKA Ultra-Turrax T25) 5 min
⇩
Addition of 5 g Quechers-Mix
(Sodium chloride + magnesium chloride (1:4)
⇩
Ultrasonic treatment (Sonorex Super 10P, Bandelin)
⇩
Centrifugation (Heraeus Megafuge 1.0) 5 min with 5.000 rpm at RT
⇩
Transfer an aliquot of supernatant (2 mL) in a new eppendorf – tube
⇩
Aliquots reduce to dryness with N2 (room temperature)
⇩
Redissolve with internal Standard in acetonitrile / water (1/1)
⇩
Briefly vortex
⇩
Filtration through syringe filter (0.2 µm PTFE)
⇩
Storage at - 80°C until measurement

Table S2. TP – analysis: Configuration of LC-MS/MS

**Liquid chromatography**	**Agilent 1290 Infinity II**		
Autosampler temperature	10 °C		
Injection volume	5 µL		
Syringe rinse	100 µL		
Analytical column	Agilent Zorbax Eclipse C18 (2.6 µm, 50 mm, 1.8 mm i.d.)		
Column temperature	40 °C		
Mobile phase A	Water + 1mmol NH_4F		
Mobile phase B	Methanol / ACN (65/35)		
Gradient program	Time (min)	A (%)	B (%)
	0.0	98	2
	0.5	98	2
	3.0	2	98
	4.0	2	98
	4.1	98	2
	4.0	98	2
Flow rate	500 µL/min		
**Mass spectrometer**	**QTRAP 6500+ (SCIEX)**		
Mode	positive ESI		
Ion spray potential	5500 V		
Source temperature	400 °C		
Scan type	Multiple Reaction Monitoring		
Dwell time	50 ms		
Software	Analyst 1.7.1		
Quantification	relative peak area		

Table S3. TP analysis - LC-MS/MS - MRM-conditions of the analyte, surrogate and internal standard (precursor (Q1) and product ions (Q3) in m/z and declustering potential (DP), entrance potential (EP), collision energy (CE) and cell exit potential (CXP) in V).

**Q1 Mass**	**Q3 Mass**	**Analyte**	**DP**	**EP**	**CE**	**CXP**
	**Positive mode**					
221	179	Atrazine-d5 (IS)	21	10	25	2.5
381	364	Triptolide-d3 (SURR)	26	10	17	18
361	115	Triptolide 1	136	10	101	14
378	361	Triptolide 2	26	10	17	18

Table S4. TP – analysis: Confirmation by Enhanced Product Ion spectra.

	**EPI (enhanced product ion spectra)**		
**Mass range**	**DP**	**EP**	**CE**
(m/z)		(V)	
50 – 450 m/z	-50	-10	-30 (±15)

Table S5. TP – analysis: Validation - Recovery (REC) and relative standard deviation (RSD) with pig liver (n = 5); in control samples (n = 2) all not detected; n. d. (not detected) = < Reporting Limit (RL).

Analyte	10 ng/g		100 ng/g	
	REC	RSD	REC	RSD
Triptolide	78	7	87	6
Triptolide-d3	75	5	80	8

Table S6. TP – analysis: Validation - Recovery (REC) of the surrogate triptolide-d3, over all trial liver and testicle samples + relative standard deviation (RSD) with 5 ng/sample wet weight.

Triptolide-d3 in trial samples	n	REC	RSD
		%	
Liver	60	81	11
Testicle	45	70	24

Table S7. VCD – analysis: Workflow of sample preparation.

Defrosting of liver samples, and weigh in pp - tubes
⇩
Spike with surrogates (50 µl)
⇩
Addition of 950 µL acetonitrile and

1000 µL sodium chloride saturated water
⇩
Homogenisation (IKA Ultra-Turrax T25) 5 min
⇩
Ultrasonic treatment (Sonorex Super 10P, Bandelin) 10 min
⇩
Centrifugation (Heraeus Megafuge 1.0) 10 min with 5.000 rpm
⇩
Transfer an aliquot of supernatant (2 mL) in a new eppendorf – tube
⇩
Addition of internal standard solution
⇩
Briefly vortex
⇩
Filtration through syringe filter (0.2 µm PTFE)
⇩
Storage at - 80°C until measurement

Table S8. VCD – analysis: Configuration of GC-MS.

**Gas chromatography**	**Trace GC Ultra (Thermo Scientific)**
Autosampler temperature	10 °C
Injector type	Split/Splitless
Injector temperature	210 °C
Injection technique	Splitless (0-3 min) wSurge (200 kPa, 1.5 min)
Injection volume	1 µL
Analytical column	ZB-624-plus (1.4 µm, 30 m, 0.25 mm i.d.)
Carrier gas	He 5.0 / 1.2 ml/min (const flow)
Column temperature	70°C (2') > 10°C/min > 280°C (3')
**Mass spectrometer**	**TSQ Quantum GC XLS(Thermo Scientific)**
Mode	EI (electron impact ionisation)
Source temperature	240°C
Transfer line temperature	275°C
Scan type	Full scan (30-650 m/z)
Software	Xcalibur 4.3.73.11
Quantification	Relative peak area

Table S9. VCD – analysis: GC-MS – retention time and detected masses of analytes used for identification and quantification of the analyte, surrogate and internal standard on quadrupole 1.

**Analyte**	**Retention time (min)**	**Q1 Mass (m/z)**
4-Vinylcyclohexene dioxide	13.5 /13.6	Σ 67, 79, 81
Piperitone (SURR)	13.9	Σ 82, 95, 110
cis-Cyclodecene (SURR)	11.3	Σ 67, 81, 54
2-Cyclohexen-1-one (IS)	8.2	Σ 68, 96

Table S10. VCD – analysis: Validation – Recovery (REC) and relative standard deviation (RSD) of 4-vinylcyclohexene dioxide and the surrogates cis-cyclodecene and piperitone in calf liver (control samples (n = 2) all not detected) for method development. The recovery of the surrogates, over all trial liver samples + relative standard deviation (RSD) were done with 1000 ng/g wet weight.

**Analyte**	**1000 ng/g**		
	**n**	**REC**	**RSD**
		%	
4-Vinylcyclohexene dioxide	5	110	15
cis-Cyclodecene in recovery	5	106	7
Piperitone in recovery samples	5	141	8
cis-Cyclodecene in trial samples	60	84	15
Piperitone in trial samples	60	113	21
